# LINCing Nuclear Mechanobiology With Skeletal Muscle Mass and Function

**DOI:** 10.3389/fcell.2021.690577

**Published:** 2021-07-21

**Authors:** Maria J. A. van Ingen, Tyler J. Kirby

**Affiliations:** ^1^Biomolecular Sciences, Faculty of Science, Vrije Universiteit Amsterdam, Amsterdam, Netherlands; ^2^Department of Physiology, Amsterdam Cardiovascular Sciences, Amsterdam Movement Sciences, Amsterdam UMC, Amsterdam, Netherlands

**Keywords:** mechanotransduction, nucleus, LINC complex, muscle mass, muscle adaptation, nuclear lamina, nesprins

## Abstract

Skeletal muscle demonstrates a high degree of adaptability in response to changes in mechanical input. The phenotypic transformation in response to mechanical cues includes changes in muscle mass and force generating capabilities, yet the molecular pathways that govern skeletal muscle adaptation are still incompletely understood. While there is strong evidence that mechanotransduction pathways that stimulate protein synthesis play a key role in regulation of muscle mass, there are likely additional mechano-sensitive mechanisms important for controlling functional muscle adaptation. There is emerging evidence that the cell nucleus can directly respond to mechanical signals (i.e., nuclear mechanotransduction), providing a potential additional level of cellular regulation for controlling skeletal muscle mass. The importance of nuclear mechanotransduction in cellular function is evident by the various genetic diseases that arise from mutations in proteins crucial to the transmission of force between the cytoskeleton and the nucleus. Intriguingly, these diseases preferentially affect cardiac and skeletal muscle, suggesting that nuclear mechanotransduction is critically important for striated muscle homeostasis. Here we discuss our current understanding for how the nucleus acts as a mechanosensor, describe the main cytoskeletal and nuclear proteins involved in the process, and propose how similar mechanoresponsive mechanisms could occur in the unique cellular environment of a myofiber. In addition, we examine how nuclear mechanotransduction fits into our current framework for how mechanical stimuli regulates skeletal muscle mass.

## Introduction

Skeletal muscle cells have the remarkable ability to adapt their size and force-generating capacity in response to changes in mechanical load. As a response to mechanical stimuli, skeletal muscle cells alter their protein metabolism primarily by modulating protein synthesis rates, with the importance of protein synthesis in determining muscle mass being well documented (Bodine et al., 2001; Marcotte et al., 2015; You et al., 2019). The mechanosensitive biochemical signaling pathways that regulate protein synthesis, such as mTORC1, MAPK, WNT/β-catenin, and YAP/TAZ, have been studied intensively over the past years and have been summarized in several excellent reviews (Bamman et al., 2018; Watt et al., 2018; Sartori et al., 2021). Our framework for how mechanotransduction, the conversion of mechanical forces into a cellular response, controls skeletal muscle mass is primarily through these biochemical signaling events. Traditionally the cell nucleus has been viewed as a passive organelle, simply serving as a reservoir for DNA and requiring cytosolic events to dictate nuclear responses. However, recent evidence has emerged showing that the nucleus itself can act as a mechanosensitive element, directly translating mechanical forces into a cellular response (Kirby and Lammerding, 2018; Aureille et al., 2019; Stephens et al., 2019) in a process termed “nuclear mechanotransduction.” The mechanisms by which nuclear mechanotransduction impacts cellular processes include nuclear envelope stretching (Enyedi et al., 2016; Lomakin et al., 2020; Venturini et al., 2020), modification of nuclear envelope proteins (Guilluy et al., 2014), histone modifications and chromatin architecture (Le et al., 2016; Nava et al., 2020), transcription factor localization (Elosegui-Artola et al., 2017; Cosgrove et al., 2021), and gene expression (Tajik et al., 2016). Limited evidence exists for a putative role of nuclear mechanotransduction in regulating muscle homeostasis and adaptation (Piccus and Brayson, 2020; Jabre et al., 2021), despite the clear link between mechanical loading and skeletal muscle mass. Much of the evidence linking nuclear mechanotransduction to skeletal muscle function comes from the study of genetic diseases, where mutations in key proteins involved in nuclear mechanotransduction result in severe dystrophic phenotypes primarily affecting skeletal and cardiac muscle (Puckelwartz et al., 2010; McGlory and Phillips, 2015; Zhou et al., 2017; Piekarowicz et al., 2019; Heller et al., 2020). While these tissue-specific disease phenotypes suggest that nuclear mechanotransduction may be important in the context of normal muscle physiology, we still have limited knowledge regarding if and how myonuclei respond to the various mechanical forces present in skeletal muscle, and how this might integrate with other well-characterized mechanosensitive signaling cascades to ultimately control muscle mass and function. In this review, we will highlight the complexes involved in nuclear mechanotransduction, examine the latest evidence for nuclear mechanotransduction in cellular adaptation, and propose mechanisms for how nuclear mechanotransduction could play a role in the regulation of muscle mass.

## Complexes Involved in Nuclear Mechanotransduction

Recent evidence demonstrates that a nucleus is able to “sense” mechanical forces and elicit various biological responses (Kirby and Lammerding, 2018; Janota et al., 2020; Lomakin et al., 2020; Venturini et al., 2020). Mechanical forces can be transmitted to the nucleus from the exterior environment through cellular adhesion complexes (Maniotis et al., 1997; Tajik et al., 2016), intracellular generated forces (Earle et al., 2020), physical compression (Lomakin et al., 2020; Venturini et al., 2020), or osmotic changes (Enyedi et al., 2016; Petridou et al., 2017). Thus, one important consideration is that differences in force application may dictate the specificity of the response. The cytoskeleton is made up out of three main polymers; actin filaments, microtubules and intermediate filaments. Together, they organize the contents of the cell, enable organelle movement, dictate the cells’ motility and shape, and connect the cell physically and biochemically to the external environment. One mechanism by which mechanical forces are transduced to the nucleus is through an intercellular network that physically connects the cytoskeleton to the nucleoskeleton via the LInker of Nucleoskeleton and Cytoskeleton (LINC) complex (Guilluy et al., 2014; Hao and Starr, 2019; Janota et al., 2020; Wong et al., 2021). The significance of this physical connection is that mechanical signals can propagate at speeds 12.5–25 times faster than passive diffusion or molecular motor-based signaling (Maurer and Lammerding, 2019), facilitating an extremely rapid cellular response. The LINC complex is a group of proteins that transverse through the nuclear envelope (NE), forming a bridge between the cytoskeleton and the nucleoskeleton (Lombardi et al., 2011; Hao and Starr, 2019). The LINC complex consists of two classes of proteins; the Klarsicht/ANC-1/SYNE homology (KASH) domain-containing proteins and the Sad-1 and UNC-84 (SUN) domain-containing proteins. The KASH family of proteins is composed of six members: nesprin-1 (encoded by *SYNE1*), nesprin-2 (encoded by *SYNE2*), nesprin-3 (encoded by *SYNE3*), nesprin-4 (encoded by *SYNE4*), Jaw1/LRMP (encoded by *JAW1*), and KASH5 (encoded by *KASH5*) (Horn et al., 2013b; Rajgor and Shanahan, 2013; Kozono et al., 2018; Janin and Gache, 2018; Zhou et al., 2018). Nesprins localize to the outer nuclear membrane (ONM) and interact with SUN proteins in the perinuclear space (PNS). Multiple nesprin-1 and -2 isoforms can be generated through alternative transcription and splicing, with the so-called giant isoforms interacting with actin via their calponin homology (CH) domain and/or microtubules via a LEWD motif-kinesin-1 interaction (Wilson and Holzbaur, 2015). In addition, smaller isoforms can interact with microtubules via various interacting partners, including AKAP450 (aka AKAP9) (Gimpel et al., 2017; Janin and Gache, 2018). Nesprin-3 interacts with intermediate filaments via the cytoskeletal linker protein plectin (Wilhelmsen et al., 2005; Wiche et al., 2015). In the case of striated muscle, the major intermediate filament protein is desmin (Heffler et al., 2020) and the major nuclear-associated plectin isoform is plectin-1 (Staszewska et al., 2015). Nesprin-4 interacts with microtubules via kinesin-1; however, its expression is restricted mainly to secretory epithelia (Roux et al., 2009) and outer hair cells of the inner ear (Horn et al., 2013a). The SUN family of proteins comprise five family members; of these, SUN1 and SUN2 are the most widely expressed (Malone et al., 1999; Crisp et al., 2005). SUN proteins form a trimeric complex that span the inner nuclear membrane (INM) and into the PNS, where their SUN domain interacts with the C-terminal KASH domain of the nesprins (Jahed et al., 2019). The N-terminus of the SUN proteins associates with nucleoplasmic structures, including the nuclear lamina (Crisp et al., 2005; Haque et al., 2006) and chromatin (Horn et al., 2013b).

Within the INM reside members of the LAP2-emerin-MAN1 (LEM) domain family of proteins (Barton et al., 2015). Of these, emerin is the most studied in the context of skeletal muscle biology, due to mutations in emerin giving rise to Emery Dreifuss muscular dystrophy (Heller et al., 2020). Emerin can bind a chromatin-interacting protein named barrier-to-autointegration factor (BAF) (Samson et al., 2018), small isoforms of nesprin-1 and nesprin-2 (Mislow et al., 2002; Zhang et al., 2005; Wheeler et al., 2007), and SUN proteins (Haque et al., 2010). Additionally, emerin binds to the nuclear lamina, enabling it to retain chromatin close to the NE during cell interphase (Berk et al., 2013; Samson et al., 2018). Furthermore, due to the physical connection to the LINC complex (Haque et al., 2006) and LEM domain proteins (Barton et al., 2015), the nuclear lamina serves as one of the major integration sites for nuclear mechanotransduction. The nuclear lamina is a filamentous meshwork of A-type lamins (lamins A and C) and B-type lamins (lamins B1 and B2). The lamina lies just underneath the INM and interacts with LEM domain proteins (Gesson et al., 2014), nuclear pore complexes (NPCs) (Xie et al., 2016), transcription factors (Ivorra et al., 2006), and chromatin through lamina-associated domains (LADs) present in the genome (van Steensel and Belmont, 2017). The nuclear lamina, along with heterochromatin, provides mechanical stability to the nucleus (Lammerding et al., 2006; Stephens et al., 2017), and the expression of lamin A scales with tissue stiffness (Swift et al., 2013). Collectively, the cytoskeleton - LINC complex - lamina - chromatin interaction network can serve as a powerful mechanism to convert mechanical signals into a cellular response.

**FIGURE 1:**
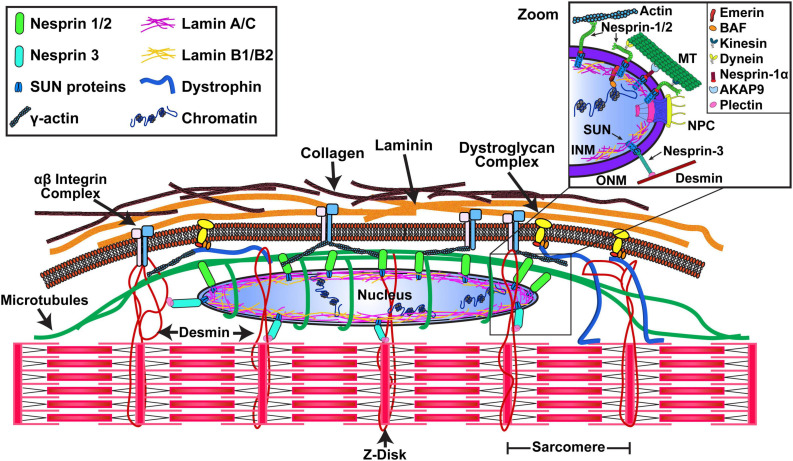
Cytoskeletal organization around myonuclei provides a mechanism for nuclear mechanotransduction. Cytoskeletal proteins (microtubules, desmin, and γ-actin) are directly connected to myonuclei via the LINC (Linker of Nucleus and Cytoskeleton) complex. The LINC complex is composed of nesprin and SUN proteins, with SUN proteins interacting with the nuclear lamina (composed of A- and B-type lamins). Chromatin is tethered to the periphery of the nucleus via the nuclear lamina, providing a mechanism for cytoskeletal mechanical signals to be transmitted directly to the nuclear interior and chromatin. Inset: The SUN components of the LINC complex interact with NPCs. Emerin binds to the nuclear lamina and the chromatin-interacting protein BAF. The giant isoforms of Nesprin-1/2 interact with actin through their CH domain and microtubules via kinesin-1 and dynein (Zhu et al., 2017), while Nesprin-1α uses AKAP9. Nesprin-3 binds to desmin via plectin. Modified from Kirby (2019).

The composition and function of the LINC complex have been studied extensively in mononucleated adherent cells, including fibroblasts, endothelial cells, and enucleated mammalian cells (cytoplasts) (Lombardi et al., 2011; Anno et al., 2012; Graham et al., 2018; Bouzid et al., 2019). Skeletal muscle cells have a unique, highly structured cytoskeletal organization, specifically designed for high force generation. Thus, the nucleo-cytoskeletal interactions in myofibers need to be arranged to accommodate this specialized cellular function (Figure 1). Moreover, myofibers are multinucleated syncytial cells with an ordered arrangement of hundreds of myonuclei located on the periphery of the myofiber, with additional myonuclei being added during muscle growth via the fusion of satellite cells (Snijders et al., 2015; Murach et al., 2017). The importance of the LINC complex in skeletal muscle has been primarily examined for its role in nuclear movement and organization during myogenesis (Zhang et al., 2010; Gimpel et al., 2017; Stroud et al., 2017); however, it may have different functions in fully mature myofibers. For example, Nesprin-1α2 associates with kinesin-1 at myotube outer nuclear membranes (Gimpel et al., 2017), but is restricted to neuromuscular junction nuclei in adult muscle (Holt et al., 2019). The current body of research about the organization and importance of the LINC complex in adult striated muscle cells comes from recent studies in cardiomyocytes (Heffler et al., 2020); however, cardiomyocytes are either mono- or bi-nucleated, with the nucleus located in the center of the cell (Janin and Gache, 2018). In cardiac cells, microtubules interact with the nucleus via AKAP6 and AKAP9 (Vergarajauregui et al., 2020); AKAP9 serves an additional function in skeletal muscle cells, where it is required for microtubule-mediated nuclear migration (Gimpel et al., 2017). Interestingly, another microtubule-organizing protein, pericentriolar material 1 (PCM1), is enriched on myonuclei in adult muscle (Winje et al., 2018). Recently, Liu et al. (2020) discovered that an alternatively spliced version of Cardiac Islet-1 Interaction Protein (CIP) interacts with several LINC complex proteins and plays a role in microtubule-mediated nuclear movement during differentiation. However, the functional role of PCM1 and CIP in myonuclear force transmission in adult muscle is still not known.

Microtubules play an important role in mechanotransduction in cardiac cells (Kerr et al., 2015; Vergarajauregui et al., 2020), where they form a cage-like structure around the nucleus. Similar cage-like structures are observed in skeletal muscle cells (Becker et al., 2020; Earle et al., 2020), although the significance of these microtubule-myonuclear interactions on nuclear morphology and mechanotransduction requires additional investigation. Similarly, the desmin - plectin - nesprin-3 interlinkage (Wilhelmsen et al., 2005; Ketema et al., 2007; Wiche et al., 2015) appears to play a role in maintaining nuclear morphology (Heffler et al., 2020) and mechanotransduction (Palmisano et al., 2015; Staszewska et al., 2015), making this complex an intriguing candidate in the context of skeletal muscle adaptation. An important consideration is that the arrangement of the LINC complex, nuclear lamina, and NE could deviate between cardiac and skeletal muscle – and even more distinctively compared to previously studied non-muscle cell-lines. We suggest that this warrants additional investigations into how the nucleus interacts with cytoskeleton and other organelles (ER, golgi, etc.) in skeletal muscle cells. Moreover, it is still not clear how newly acquired myonuclei integrate into the highly complex and ordered cytoskeletal network and what LINC complex reorganization must occur to facilitate this process. Finally, the contribution of specific proteins in nucleo-cytoskeletal coupling will have to be considered when determining what forces may be transmitted to myonuclei during passive (stretch) and active (contractile) force generation.

## Evidence for Nuclear Mechanotransduction in Non-Skeletal Muscle Cells

The first evidence of nuclear mechanotransduction came in the 1990s, when pioneering work by the Ingber laboratory showed that nuclei are “hard-wired” to their surrounding cytoplasm and that forces applied to either integrins or the cytoskeleton could elicit a physical response from the nucleus (Maniotis et al., 1997). Since those seminal studies, significant work has gone into trying to dissect which cellular responses to mechanical signals can be attributed to direct responses by nuclei themselves. Supporting evidence for the importance of nuclear mechanics come from disease-causing mutations in proteins involved in nuclear mechanotransduction (Piekarowicz et al., 2019; Donnaloja et al., 2020; Heller et al., 2020). Though the underlying molecular mechanisms by which these proteins regulate tissue homeostasis have not yet been fully elucidated, nuclear mechanotransduction has been shown to induce chromatin rearrangement (Le et al., 2016; Nava et al., 2020), NE-unfolding (Enyedi et al., 2016; Lomakin et al., 2020; Venturini et al., 2020), the post-translational modification of nuclear proteins (Guilluy et al., 2014), transcription factor translocation (Elosegui-Artola et al., 2017), and gene expression (Tajik et al., 2016).

### Chromatin Stretching/Modifications

One proposed mechanism for how mechanical forces transmitted to the nucleus can lead directly to a cellular response is through changes in chromatin organization or accessibility to transcription factors. Condensed chromatin, or heterochromatin, that is localized to the nuclear periphery is often adjacent to and makes contact with the nuclear lamina at LADs (van Steensel and Belmont, 2017). DNA is wrapped around histones, forming tightly compacted heterochromatin, inaccessible for transcription, along with less condensed euchromatin (Janssen et al., 2018). Moreover, A-type lamins, in cooperation with LEM-domain proteins, are critical for tethering heterochromatin to the NE (Solovei et al., 2013); thus, these physical interactions between chromatin and the nuclear lamina can allow forces to be transmitted to chromatin. To this end, Tajik et al. (2016) showed that local surface force at the plasma membrane, acting through the LINC complex, can directly stretch the chromatin and induce a rapid increase in transcription. This force-induced chromatin stretching and transcriptional upregulation is sensitive to both levels of H3K9me3 and force application frequency, where low H3K9me3 levels and low frequency are required for sufficient chromatin stretching and subsequent recruitment of RNA-polymerase II (Pol II) (Sun et al., 2020). Thus, epigenetic alterations to chromatin may serve as a mechanism for determining the specificity of the transcriptional response to nuclear force transmission. Recently, Nava et al. (2020) found that stretching of the nuclear membrane results in the rapid loss of histone methylation at H3K9 and H3K27, producing a more deformable nucleus that is protected from force-induced damage. Intriguingly, prolonged mechanical strain leads to large-scale chromatin rearrangements and the replacement of H3K9me2,3 with H3K27me3, leading to global transcriptional silencing during stem cell commitment (Le et al., 2016); however, it is unclear if a similar mechanism would occur in a terminally differentiated cell. The frequency and duration of force application on the nucleus appear to be important for determining the change in chromatin organization.

### Transcription Factor Localization/Nuclear Pores

It is suggested that transmission of mechanical forces between the cytoskeleton and nucleoskeleton could directly influence the transport of proteins across the NE through NPCs (Donnaloja et al., 2019). NPCs have been shown to interact with both the nuclear lamina (Al-Haboubi et al., 2011; Xie et al., 2016; Kittisopikul et al., 2021) and SUN1 (Liu et al., 2007), providing a mechanism for nuclear force transmission to alter NPC conformation. Work from the Roca-Cusachs laboratory demonstrated that direct application of force to the nucleus causes nuclear deformation, thereby stretching the NPC and allowing for increased import of YAP into the nucleus (Elosegui-Artola et al., 2017). Similarly, YAP nuclear entry in response to cyclic strain is impaired when the LINC complex is disrupted in mesenchymal stem cells, (Driscoll et al., 2015), indicating that nuclear mechanotransduction can regulate YAP localization in response to mechanical cues. In addition to altering the permeability of NPCs, mechanical signals can alter NPC localization through the redistribution of LINC complex proteins (Hoffman et al., 2020). The dogma of the NPC being a highly rigid structure has been challenged in recent years (Knockenhauer and Schwartz, 2016; Pulupa et al., 2020). One intriguing hypothesis is that repeated mechanical signals result in the clustering of NPCs at the site of force transmission and physically influence NPC conformation to affect nuclear transport.

Additionally, it has become evident that INM proteins influence the nucleo-cytoplasmic flux of transcription factors. The transcription factor β-catenin has been shown to be associated with nuclear envelope proteins such as emerin, lamins A/C and the LINC complex (Markiewicz et al., 2006; Tilgner et al., 2009; Neumann et al., 2010; Uzer et al., 2018). Uzer and colleagues found that disabling the LINC complex via co-depletion of SUN1/2 impedes the nuclear entry of β-catenin by limiting its nesprin-mediated interaction with the NE (Uzer et al., 2018). Since the LINC complex plays a critical part in the transmission of applied mechanical force from the cellular surface to the nucleus, Uzer et al., propose a new pathway by which LINC complex-mediated connectivity may play a role in signaling pathways that depend on the nuclear entry of β-catenin. In addition to the LINC complex, emerin contributes to the regulation of the β-catenin nuclear flux by binding to cytoplasmic β-catenin to restrict it from the nucleus (Markiewicz et al., 2006). To further investigate the relationship between β-catenin and nuclear β-catenin-binding partners, Tilgner et al. (2009) performed a study focusing on the expression of emerin, A-type lamins, and peroxisome proliferators-activated receptors γ (PPARγ) in preadipocytes and dermal fibroblasts. The authors found that the expression of NE proteins, A-type lamins, and emerin is directly linked to the balance between β-catenin and the PPARγ signaling to control the adipogenic capacity of the cell. Collectively, nuclear mechanotransduction can influence transcription factor localization to the nucleus, either through direct interactions or by modulating transport across the NPC.

### NE and ER Unfolding

Another mechanism through which the nucleus responds to external forces is via the unfolding and stretching of the NE. Recently, Lomakin et al. (2020) found that migrating immune cells unfold and stretch their NE to adapt to environmental confinement and that enucleated cells show less motility in similar circumstances. Venturini et al. (2020) support these findings, with the demonstration of nuclear deformation due to confinement of primary progenitor stem cells, which leads to INM unfolding and intracellular spatial positioning of the nucleus. Mechanistically, NE stretching leads to the release of calcium; this activates calcium-dependent cytosolic phospholipase A2 (cPLA2), which catalyzes the formation of arachidonic acid to ultimately regulate myosin-II activity (Lomakin et al., 2020; Venturini et al., 2020). This mechanism of nuclear stretch-activation of cPLA2 was first identified by the Niethammer group in response to tissue damage (Enyedi et al., 2016). Similarly, deformation of nuclei with high membrane tension triggers Ca^2+^ release from the ER to modulate chromatin methylation levels (Nava et al., 2020). Collectively, NE stretch-dependent Ca^2+^ release is emerging as a powerful intermediary between mechanical inputs and cellular responses. In addition to the effect on calcium release, the amount of NE folding or “wrinkling” is associated with the translocation of mechanosensitive transcription factors, including YAP/TAZ (Cosgrove et al., 2021). One explanation for this altered transcription factor localization could be the accumulation of NPCs in NE invaginations, as has been shown in progeroid cells (Goldman et al., 2004; Röhrl et al., 2021), leading to a physical barrier affecting NPC transport. Lastly, nuclear force transmission has been shown to alter the assembly of the network of A-type lamins, exposing epitopes that are involved in chromatin interactions (Ihalainen et al., 2015). Thus, the extent of NE stretching will be highly dependent on the ability of forces to deform the nucleus, a process determined by the mechanical properties of the nucleus (Lammerding et al., 2006; Stephens et al., 2017).

### Biochemical

Finally, mechanical signals lead to post-translational modifications of INM proteins and A-type lamins resulting in changes to the mechanical properties of the nucleus (Swift et al., 2013; Buxboim et al., 2014; Guilluy et al., 2014; Guilluy and Burridge, 2015; Graham et al., 2018; Gilbert et al., 2019; Ikegami et al., 2020). In isolated mammalian nuclei, pulses of force applied to nesprin 1 result in a decrease in nuclear strain, indicating local nuclear stiffening (Guilluy et al., 2014). The authors found that neither chromatin nor nuclear actin were involved in force response; however, emerin becomes tyrosine phosphorylated by tyrosine kinase Src as a reaction to applied force, strengthening the connection between A-type lamins and the LINC complex. In addition to phosphorylation of emerin, phosphorylation of lamins is a well-known mechanism involved in nuclear lamina assembly and disassembly in cell division. During interphase, A-type lamins phosphorylation is low, allowing for network assembly beneath the INM, whereas phosphorylation results in a shift toward nucleoplasmic localization (Buxboim et al., 2014; Kochin et al., 2014; Ikegami et al., 2020). Studies show that phosphorylation of A-type lamins on Ser22 is associated with soft matrix (Swift et al., 2013), and that their dephosphorylation is caused by myosin-II activity and matrix stiffness (Buxboim et al., 2014). Further experimentation revealed that the phosphodynamics of Ser22 are critical in determining the structural organization and mechanics of nuclei during cell spreading (Buxboim et al., 2014). The precise mechanism by which mechanical forces can modulate phosphorylation of nuclear envelope proteins remains under investigation, including whether this process is regulated by altering kinase activities or accessibility of the kinase to cryptic phosphorylation sites within their protein substrates. Nevertheless, the observed mechanically induced phosphorylation implicates a structural role for phosphorylation in mechanotransduction through control of nuclear stiffening and nucleo-cytoskeletal coupling (Maurer and Lammerding, 2019).

## Potential Mechanisms for Nuclear Mechanotransduction in Regulating Muscle Mass and Function

Many structural adaptations occur in skeletal muscle that give rise to changes in muscle mass (Jorgenson et al., 2020), with mechanical loading being a primary driver. Despite this clear association, further work is necessary to determine how structural changes in the muscle may provide feedback to alter the mechanosensitivity of the tissue. Muscle mass is largely governed by protein synthesis rates (Joanisse et al., 2020). However, studies using genetic approaches to manipulate protein turnover and increase muscle mass have failed to demonstrate a concomitant change in force output (Graber et al., 2019; Hunt et al., 2021), suggesting that mechanical input is critical for functional adaptations. To this end, mechanical signals may be important for creating a transcriptional profile that is permissive for changes in muscle mass and function (Phillips et al., 2013; Stokes et al., 2020). Therefore, force transmission to the nucleus could serve as an important regulatory mechanism for altering chromatin organization and transcription factor localization/activity in skeletal muscle cells.

**FIGURE 2:**
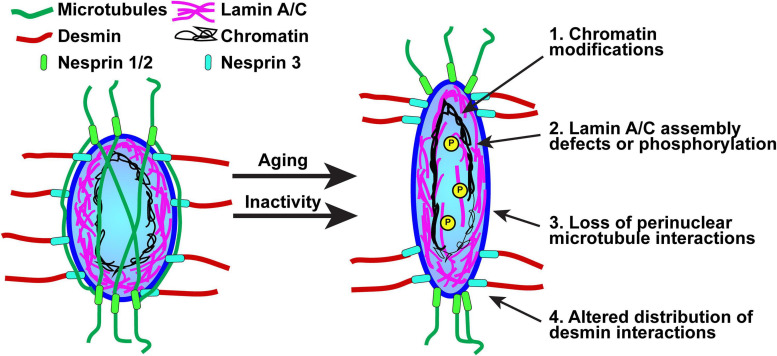
Proposed mechanisms for nuclear-intrinsic and -extrinsic changes that could alter nuclear morphology in response to inactivity or aging. Nuclear stiffness is determined by both A-type lamins expression/assembly and chromatin modifications, specifically the amount of heterochromatin. Phosphorylation of A-type lamins leads to a nucleoplasmic localization and a decrease in nuclear stiffness. Nuclear morphology is also influenced by cytoskeletal forces acting through the LINC complex. Microtubules form a cage-like structure around the nucleus and provide compressive force; thus, elongation could be driven by a decrease in microtubule-nuclear interactions. Loss of desmin or plectin-1 results in more rounded nuclei, suggesting that changes in either the number or arrangement of desmin-nuclear interactions could elicit a change in nuclear morphology.

Mislocalization of myonuclei is associated with cellular dysfunction and a range of muscle diseases. An intact LINC-complex is critical for the localization of the nuclei in skeletal muscle development (Gundersen and Worman, 2013; Janota et al., 2020). For example, nesprin-1 is required for myonuclear anchoring in skeletal muscle (Zhang et al., 2010; Stroud et al., 2017). Double knock-out mouse models of nesprin-1 and nesprin-2 show a cardiomyopathy phenotype alongside changes in nuclear deformation and chromatin decondensation (Banerjee et al., 2014). Moreover, the loss of SUN1 and SUN2 in knockout mouse models demonstrated that nuclear positioning in skeletal muscle cells is disrupted (Lei et al., 2009). Conditional deletion of LAP1, an emerin-interacting protein, causes muscular dystrophy in mice, suggesting that this emerin binding partner is essential for skeletal muscle maintenance and postnatal skeletal muscle growth (Shin et al., 2013, 2014, 2017). In addition to genetically induced disruption of nuclear position, changes in myonuclear morphology have been observed following chronic resistance exercise followed by detraining (Murach et al., 2020) and physiological aging (Brack et al., 2005; Bruusgaard et al., 2006; Cristea et al., 2010), suggesting that nuclear stability and/or nucleo-cytoskeletal coupling may adapt to changes in mechanical signals. It remains unclear whether this change in morphology is the result of intrinsic myonuclear adaptations or altered LINC complex connections and cytoskeletal forces (Figure 2). Moreover, how this change affects the mechanoresponsiveness of the myonucleus is unknown, as are the potential implications for myofiber homeostasis and adaptation, including nuclear mechanotransduction potentially coordinating a transcriptional profile that complements proteins synthesis changes to influence myofiber structure and function.

It remains to be determined whether mechanoresponsive protein synthetic pathways such as mTORC1, YAP/TAZ, and β-catenin are influenced by nuclear mechanotransduction in mature muscle cells, and if so, how these processes might synergize to affect muscle function. YAP/TAZ signaling has emerged as a potent regulator of skeletal muscle mass and function (Watt et al., 2015, 2018) and adaptation (Goodman et al., 2015), and has been suggested to be involved in age-related muscle atrophy (Setiawan et al., 2021). A proteomics study revealed that the expression of YAP is ∼two fold higher in slow-twitch muscle fibers than in fast-twitch muscle fibers from young subjects; in aged subjects, the YAP expression was ∼50% lower in both muscle fiber types compared to younger controls (Murgia et al., 2017). However, YAP/TAZ signaling may be elevated in aged skeletal muscle and associated with changes in the nuclear lamina (Iyer et al., 2021).Together, these results suggest that altered YAP expression and localization via changes in nuclear architecture (Cosgrove et al., 2021) or nuclear mechanotransduction (Driscoll et al., 2015) could play a role in muscle adaptation and age-dependent loss of skeletal muscle mass. Wnt/β-catenin signaling has been suggested to be involved in augmenting myofiber hypertrophy in response to increased mechanical load (Armstrong and Esser, 2005; Armstrong et al., 2006) and may be modulated by nuclear access to β-catenin via the LINC complex (Uzer et al., 2018). The well-characterized mechanosensitive MRTF/SRF pathway was recently shown to be activated in response to muscle contractions and associated with increased protein synthesis (Solagna et al., 2020), with the nuclear retention of MRTF-A being regulated by A-type lamins and emerin (Ho et al., 2013). Lastly, the transcription factor JunB is important for maintaining skeletal muscle mass and can promote hypertrophy (Raffaello et al., 2010). JunB is a member of the AP-1 family of proteins, of which c-Jun and c-Fos have been shown to interact with A-type lamins (Ivorra et al., 2006; Ikegami et al., 2020). Thus, it would be interesting to determine whether similar interactions occur between A-type lamins and JunB in skeletal muscle, and if so, whether the interaction is modulated by mechanical inputs. Collectively, changes in transcription factor localization or activity in response to alterations in nuclear morphology or mechanics may have important implications for regulating the skeletal muscle transcriptome.

Recently, mechanical loading has been shown to induce both DNA and histone modifications in skeletal muscle (McGee and Walder, 2017; Jacques et al., 2019; Solagna et al., 2020; Walden et al., 2020). Specifically, acute mechanical overload in mice results in hypomethylation of genes known to be involved in muscle maintenance, including known regulators of mTORC1 signaling (Walden et al., 2020). Similarly, an acute bout of resistance exercise in humans increases H3K27me3 distribution at 16 loci while total H3K27me3 levels were unaffected (Lim et al., 2020). Eccentric contraction induces phosphorylation of serine 10 on histone 3 (H3S10ph) in mice, with no changes in levels of H3-K9K14ac and H3K4me3 (Solagna et al., 2020). Collectively, these results suggest that changes in mechanical load alter DNA methylation and histone modifications to facilitate a mechanosensitive transcriptional response. During physiological aging there are global changes in histone modifications, including loss of H3K9me3 (Yoshihara et al., 2019) and increased H3K27ac (Zhou et al., 2019), it is possible that age-related alterations in nuclear morphology (Brack et al., 2005; Bruusgaard et al., 2006; Cristea et al., 2010) could promote changes in global chromatin organization. Further investigations are warranted to investigate how these modifications alter the chromatin accessibility in skeletal muscle and whether nuclear mechanotransduction plays a regulatory role in the response to mechanical loading or aging.

## Important Considerations and Future Directions

The importance of protein metabolism in establishing a given level of muscle mass is clear; however, changes in protein synthesis do not always correspond to a change in muscle function, suggesting that the effect is dependent on the specific proteins being synthesized. We propose that the impact of nuclear morphology and nuclear mechanotransduction in skeletal muscle homeostasis and adaptation is an area worthy of further investigation. To date, most of the work in skeletal muscle has focused on nuclear abundance as an important determinant of myofiber size and adaptation (McCarthy et al., 2011; Murach et al., 2017; Psilander et al., 2019; Cramer et al., 2020; Hansson et al., 2020), with almost no focus on the nucleus as being a central player in the mechanotransduction response to mechanical load. The importance of nuclear mechanotransduction and the LINC complex in nuclear migration during myogenesis is well established (Zhang et al., 2010; Gimpel et al., 2017; Stroud et al., 2017), yet whether this importance persists for tissue maintenance is unclear. We still have a limited understanding of how myonuclei are integrated into the cytoskeleton in muscle fibers, how this is accomplished during the unique phenomenon of myonuclear addition, and finally, how this integration may change during adaptation, disease, or aging.

One of the primary challenges in studying the role of nuclear mechanotransduction in a specific cellular response is decoupling the mechanoresponse of the nucleus from cell surface/cytoplasmic signaling events. This can be accomplished by either performing assays on isolated nuclei (Guilluy and Burridge, 2015; Stephens et al., 2019), or via the more biologically relevant technique of restricting mechanical signals from reaching the nucleus. In practice, this can be done by expressing dominant-negative nesprin or SUN constructs (Lombardi et al., 2011; Uzer et al., 2018), which globally disrupt all LINC complexes, or via targeting of specific LINC complex or LINC-complex associated proteins (Staszewska et al., 2015; Tajik et al., 2016; Cosgrove et al., 2021).

Another significant challenge will be identifying mechanisms that control the specificity of the response. For example, if chromatin stretching can induce transcriptional activation, how can this be restricted to specific genes or loci? There may need to be additional layers of regulation, such as additional epigenetic modifications that modulate the response (Sun et al., 2020). Integrating genetic and biophysical methods with either advanced-tissue engineering approaches or novel mouse models will be necessary to study the causative effects of nuclear mechanotransduction on muscle mass and function. For the mouse models, utilizing inducible- and tissue-specific strains (Murach et al., 2020) will allow for separating developmental effects from those necessary for maintenance and adaptation during adulthood. Combining these approaches with recent advances in single-nuclear sequencing (Ding et al., 2020; Dos Santos et al., 2020; Petrany et al., 2020) and chromatin-accessibility technologies (Klein and Hainer, 2020) could allow for discovery of novel transcriptional and/or chromatin organization mechanisms. Moreover, recently developed LINC complex-based tension sensors (Arsenovic et al., 2016; Déjardin et al., 2020) could be implemented to quantify the amount of cytoskeleton-to-nucleus force transmission in muscle fibers. Lastly, as an alternative to experimental-based approaches, computational modeling has potential utility for the prediction of how various mechanical force-generating and -transmitting structures in skeletal muscle contribute to nuclear mechanobiology (Mohammed et al., 2019).

Seminal work over the past 30 years has started to unravel the molecular details for how mechanical forces are transduced in skeletal muscle in order to control tissue mass and function. Nevertheless, the potential influence of nuclear mechanotransduction on directly modulating myonuclear organization and/or activity has been largely overlooked. Additional work is necessary to understand the precise role that cytoskeletal-nuclear force transmission has on the skeletal muscle transcriptome, and if so, how specificity in controlled. Furthermore, decoupling these events from cytoplasmic signaling events remains a challenge, as it is likely that synergy between multiple mechanotransduction pathways is required to produce the appropriate response. We propose that nuclear mechanotransduction may provide an additional “fine-tuning” role for priming the muscle cell for the appropriate transcriptional response to mechanical stimuli (Tajik et al., 2016), or possibly serve to alter the mechanical properties of the nucleus to protect the genome from repeated mechanical stresses (Nava et al., 2020), ultimately serving as an integral link between mechanical loading and muscle mass regulation.

## Author Contributions

All authors listed have made a substantial, direct and intellectual contribution to the work, and approved it for publication.

## Conflict of Interest

The authors declare that the research was conducted in the absence of any commercial or financial relationships that could be construed as a potential conflict of interest.

## Abbreviations

BAF: barrier-to-autointegration factor
CIP: cardiac Islet-1 interaction protein
CH: calponin homology domain
cPLA2: phospholipase A2
ECM: extracellular matrix
ER: endoplasmic reticulum
INM: inner nuclear membrane
KASH, Klarsicht, ANC-1: Syne homology
LAD: lamina-associated domain
LEM: LAP2-emerin-MAN1 domain
LINC: linkers of nucleoskeleton and cytoskeleton
NE: nuclear envelope
nesprin: nuclear envelope spectrin repeat protein
NPC: nuclear pore complex
ONM: outer nuclear membrane
PCM1: pericentriolar material 1
PNS: perinuclear space
Pol-II: RNA-polymerase II
PPARγ: peroxisome proliferators-activated receptors γ
SUN1/2: Sad1p-UNC-84 domain 1 and 2 proteins
TAZ: transcriptional coactivator with PDZ-binding motif
YAP: Yes-associated protein.
